# InP-Substrate-Based Quantum Dashes on a DBR as Single-Photon Emitters at the Third Telecommunication Window

**DOI:** 10.3390/ma14040759

**Published:** 2021-02-05

**Authors:** Paweł Wyborski, Anna Musiał, Paweł Mrowiński, Paweł Podemski, Vasilij Baumann, Piotr Wroński, Fauzia Jabeen, Sven Höfling, Grzegorz Sęk

**Affiliations:** 1 Laboratory for Optical Spectroscopy of Nanostructures, Department of Experimental Physics, Faculty of Fundamental Problems of Technology, Wrocław University of Science and Technology, Wybrzeże Wyspiańskiego 27, 50-370 Wrocław, Poland; anna.musial@pwr.edu.pl (A.M.); pawel.mrowinski@pwr.edu.pl (P.M.); pawel.podemski@pwr.edu.pl (P.P.); grzegorz.sek@pwr.edu.pl (G.S.); 2 Technische Physik, University of Würzburg and Wilhelm-Conrad-Röntgen-Research Center for Complex Material Systems, Am Hubland, D-97074 Würzburg, Germany; vasilij.baumann@instrunext.com (V.B.); piotr-andrzej.wronski@physik.uni-wuerzburg.de (P.W.); fauzia.jabeen@uni-wuerzburg.de (F.J.); sven.hoefling@physik.uni-wuerzburg.de (S.H.); 3 Faculty of Engineering and Physical Sciences, University of Southampton, Southampton SO17 1BJ, UK; 4 School of Physics and Astronomy, University of St. Andrews, North Haugh, St. Andrews KY16 9SS, UK

**Keywords:** single-photon emitter, III–V quantum dot, telecommunication spectral range, photonic structure, extraction efficiency

## Abstract

We investigated emission properties of photonic structures with InAs/InGaAlAs/InP quantum dashes grown by molecular beam epitaxy on a distributed Bragg reflector. In high-spatial-resolution photoluminescence experiment, well-resolved sharp spectral lines are observed and single-photon emission is detected in the third telecommunication window characterized by very low multiphoton events probabilities. The photoluminescence spectra measured on simple photonic structures in the form of cylindrical mesas reveal significant intensity enhancement by a factor of 4 when compared to a planar sample. These results are supported by simulations of the electromagnetic field distribution, which show emission extraction efficiencies even above 18% for optimized designs. When combined with relatively simple and undemanding fabrication approach, it makes this kind of structures competitive with the existing solutions in that spectral range and prospective in the context of efficient and practical single-photon sources for fiber-based quantum networks applications.

## 1. Introduction

Single-photon sources (SPS) are fundamental components of many nanophotonic devices and find applications in the field of quantum information technology. They ensure security in quantum key distribution protocols or in quantum repeaters where having a pure and efficient single-photon source is the most important element [1]. Scalable and high-volume fabrication technology of this kind of sources is of special practical importance, especially those operating in the lowest losses third telecommunication window, to employ the quantum communication schemes for long-haul optical interconnects. An ideal SPS is characterized by high purity of a single-photon emission (minimized multiphoton emission events) and high brightness. The most common type of SPS based on spontaneous parametric down conversion allow for relatively high purity of single-photon emission but with rather low brightness due to low conversion efficiency [2,3]. The reason is the probabilistic process of photon generation, which prevents reaching the requirements of on-demand single-photon emission [4,5]. Other single-photon sources based on defects in carbon nanotubes [6], atomic ions [7], nitrogen-vacancy centers in diamond [8], defects in silicon carbide [9], and gallium nitride [10] can reach desired parameters, however, they show a number of technological problems preventing their straightforward application, including their integration with existing semiconductor platforms, scalability, stability, and brightness [5,11]. SPS based on quantum dots (QDs) have one of the best purities of single-photon emission [12,13,14] and have demonstrated many desired properties as emission of entangled photon pairs [15,16] together with application possibilities also in an on-demand operation mode [17,18]. The current quantum-dot-based solutions are mostly limited by collection efficiency of emission to a first lens (or an optical fiber) of the detection/collection system [5,19], which severely hinders their applicability as efficient telecommunication SPS [11,19]. Many photonic structures with QDs have been demonstrated to improve the extraction of emission from a single QD, e.g., photonic crystal cavities [20,21], circular Bragg resonators [22,23], micropillars [17,24,25], also electrically controlled [13], microlenses [26,27,28,29,30], and mesa structures [31,32,33]. Nevertheless, the major progress and the most important milestones in this field concern QDs beyond the third telecommunication window [11,19].

For single-photon emission in the third telecommunication window and the possibility of implementing long-haul communication, two material systems for SPS based on QDs are the most promising: In(Ga)As QDs on GaAs or InP substrates. Technology based on GaAs substrate is well developed, however, demanding strain engineering is required [34] to redshift the emission to 1.55 µm range. At least several approaches have been used, e.g., utilizing the strain-reducing layer [35,36,37,38], growing on a metamorphic buffer layer [34,39,40], multistacking of QDs [41], or growth on a seeding layer [42]. For GaAs-based QD structures, single-photon emission at the third telecommunication window has been demonstrated only using a special metamorphic buffer layer grown by Metalorganic chemical vapor deposition (MOCVD) [43], with reported emission of entangled photons [44] and emission of indistinguishable photons [45], also with the possibility of precise piezo-tuning [46] and generation of single-photons on demand [47]. However, the growth process of such QDs is very demanding and prone to technological complications, deteriorating the optical quality of the final material [48], which is probably the reason for still lacking results on high-quality and high-brightness photonic structures out of that material system at 1.55 µm [11,40].

InAs/InP materials combination allows for creation of high-quality nanostructures emitting in the third telecommunication window without using sophisticated strain engineering [20,40,49,50,51]. When molecular beam epitaxy (MBE) is employed in this material system, strongly elongated quantum dots called quantum dashes (QDashes) are naturally formed [52,53]. Due to typically high surface density, they are not usually in the forefront of the SPS application and hence less explored. Despite this, some preliminary data for QDashes in the context of single-photon emission under nonresonant excitation exists [54,55]. QDashes have also been demonstrated as having some other advantageous features, e.g., prospects for spin memory [56] or single-photon emission at elevated temperatures [55]. Growth of InAs nanostructures on InP substrate by MOCVD leads to more common symmetrical quantum dots with a more straightforward control of their areal density [57], using a special “double-capping” method, single-photon emission with optical nonresonant [58] and quasi-resonant excitation [12] have been shown. Application of relatively demanding MBE growth, involving additional ripening process, brought in-plane symmetric, low-density quantum dots [50,59], for which triggered single-photon emission has also been reported [60]. On the other hand, droplet epitaxy approach enabling reduction in surface density has been presented [49], also using the MBE technology [51]. Emission of single photons with electrical excitation in the diode structure [61], as well as the possibility of obtaining entangled photons and quantum teleportation of qubits have been demonstrated for nanostructures of that kind [62].

In spite of all these proof-of-concept demonstrations, single-photon sources in the third telecommunication window based on quantum-dot-like structures still have brightness restrictions and difficulties in implementing of high-quality distributed Bragg reflectors (DBR) matched to the InP substrate what would allow for the emission extraction efficiency increase [40]. To date, only the use of an intricate horn structure appeared promising in this field, demonstrating an increase in the extraction efficiency up to 10.9% [63], i.e., a value that has not been beaten for 13 years now and hence remaining one of the main challenges. Within this work is explored experimentally, a more straightforward and technologically much less demanding photonic structure design in a form of a cylindrical or cuboidal mesa on a DBR structure underneath a layer of QDashes, which show the possibility of increasing the extraction efficiency of emission based on numerical simulations.

In this report is presented a first step towards simple approach for realization of practical single-photon emitters with multiphoton events probabilities below 5% in the third telecommunication window and based on InAs quantum dashes on a DBR grown by MBE on an InP substrate and located inside a cylindrical photonic mesa structure. This design has not yet been demonstrated for QDashes, most likely due to the strain and defect modifications introduced usually by the DBR beneath, influencing the proper growth condition of QDashes and, therefore, their internal quantum efficiency. We show that it can be a way of constructing affordable nanophotonic devices, based on unsophisticated photonic confinement structures. In addition, compatibility of our experimental data with the numerical simulations indicates the possibility of the emission extraction efficiency control, paving the way towards a more efficient SPS design in this material system.

## 2. Materials and Methods

**Figure 1:**
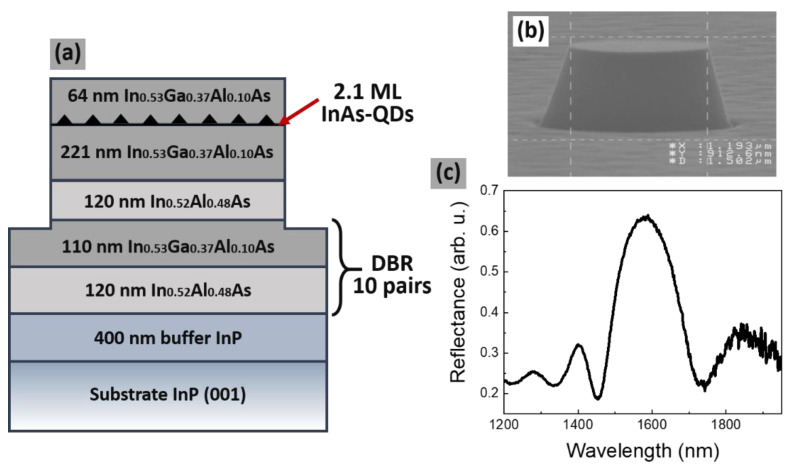
(**a**) Scheme of the investigated structure with InAs/InGaAlAs/InP elongated quantum dashes. (**b**) SEM image of the mesa structure. (**c**) Reflectivity spectrum for a planar structure at low temperature.

The investigated structure was grown by MBE on an InP (001) substrate. The sequence of epitaxial layers, shown in Figure 1a, begins with 400 nm of InP buffer layer.

Then, a distributed Bragg reflector formed of 10 pairs of In_0.52_Al_0.48_As and In_0.53_Ga_0.37_Al_0.10_As layers with thicknesses of 120 and 110 nm was grown, respectively, to obtain enhanced reflection in the third telecommunication range—see Figure 1c. A well-pronounced stop band in the target spectral range with reflectivity of about 0.65 confirms good optical quality of the DBR structure. The DBR is followed by 221 nm of In_0.53_Ga_0.37_Al_0.10_As (lattice-matched to InP) to create sufficient carrier confinement and to provide growth conditions for the elongated nanostructures formation [64]. In order to grow QDashes (by self-assembly in a Stranski–Krastanov mode), 2.1 monolayers of InAs material were deposited. The QDash layer was covered with a 64 nm thick In_0.53_Ga_0.37_Al_0.10_As layer. Significant shape anisotropy of the grown nanostructures was confirmed by the degree of linear polarization of the surface emission of about 20% (not shown here), which is a typical value for InAs QDashes on InP [65,66]. Further, by a combination of electron beam lithography and wet etching were fabricated photonic structures in a form of cylindrical mesas with different sizes, i.e., with a diameter in range from 500 to 1500 nm and a height of about 700 nm (Figure 1b).

Optical characterization was performed using a microphotoluminescence (µPL) setup providing high spatial resolution. For the dependence of emission on the excitation power was used a system equipped with a 1 m-focal length spectrometer coupled with a liquid-nitrogen-cooled InGaAs linear array detector offering, in total, spectral resolution of about 50 µeV. Nonresonant excitation was provided by a continuous wave (CW) 660 nm semiconductor laser, focused on the sample surface by a microscope objective to a beam diameter on the order of single micrometers. Time-resolved photoluminescence (TRPL), extraction efficiency, and statistics of emission events (to evaluate single-photon purity) were measured using 0.32 m-focal length monochromator as a spectral filter for selection of emission lines from a single QDash, using nonresonant excitation by a CW 787 nm laser or an 805 nm semiconductor diode laser with 80 MHz train of approximately 50 ps-long pulses. These measurements were carried out with fiber-coupled NbN superconducting nanowire single-photon detectors with approximately 50% of quantum efficiency and dark count rate of 100 cps at 1.55 µm, combined with multichannel picosecond event timer with a time bin width of 256 ps. In addition, photon autocorrelation measurements were performed in a Hanbury Brown and Twiss fiber interferometer configuration. The sample was kept in a liquid-helium continuous-flow cryostat at the temperature of about 5 K during all the measurements.

## 3. Results and Discussion

**Figure 2:**
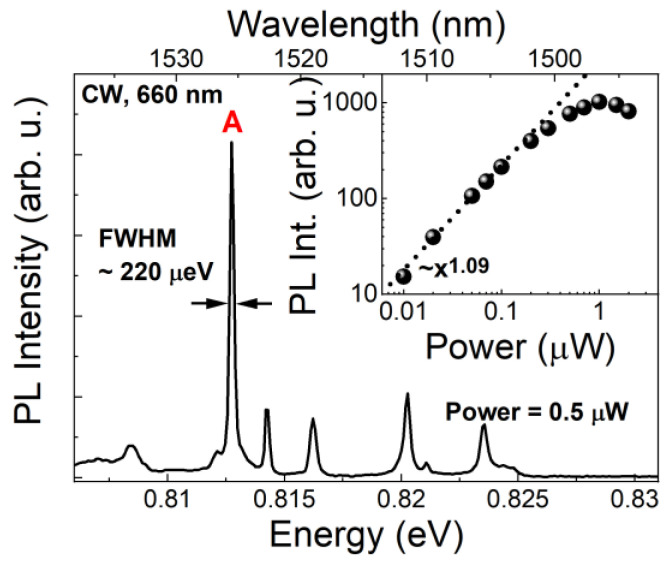
Microphotoluminescence spectrum from single quantum dashes (QDashes) under continuous-wave excitation (660 nm). Inset: spectrally integrated intensity of the line A as a function of the excitation power. The experimental data were fitted with a power function (dotted line).

Figure 2 shows a typical for these structures low temperature µPL spectrum in the third telecommunication window spectral range measured under CW nonresonant excitation (power of 0.5 µW measured outside of the cryostat).

Fabrication of the mesa structure (diameter of about 1 µm and height of ~0.7 µm in this case) enables observation of single QDash emission lines. The strongest line, marked as A (~0.8127 eV), is well isolated and is selected as a candidate for further single-photon emission study. The spectrum shown in Figure 2 is composed from a few emission lines. This is a typical µPL spectrum from self-assembled nanostructures, where excitonic complexes from one quantum dash coexist spectrally with the emission from neighboring dashes. Assuming the typical surface density for quantum dashes about 10^10^ cm^−2^ and a mesa diameter of about 1 µm, we get about 80 dashes per mesa structure. Because of the self-assembled quantum dashes’ inherent size/shape distribution on the sample surface the spectrum will differ depending on the examined spot on the sample. On the other hand, the spectral structure of excitonic complexes emission from a single quantum dash should be consistent, to some extent, with other dashes of the same type, however, this analysis is beyond the scope of this work [67], as long as a single bright line can be selected in the target spectral window. The linewidth of the line A is about 220 µeV, which is consistent with the values for QDashes observed previously [55] and mostly originates from the spectral diffusion processes predominant in the case of the nonresonant excitation scheme. The dependence of the A line intensity on the excitation power (inset in Figure 2) shows almost linear increase in the intensity with saturation for approximately 1 µW, suggesting that the A emission line is related to the radiative recombination of a neutral exciton or charged exciton (the exact identification of particular excitonic complexes is beyond the scope of this work).

**Figure 3:**
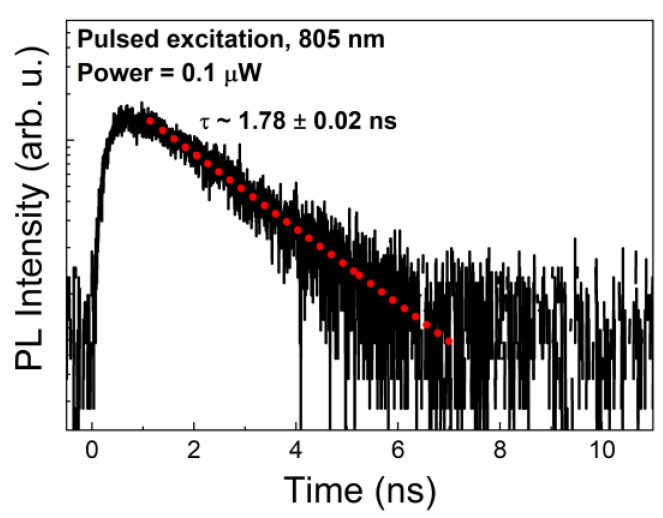
Time-resolved microphotoluminescence (µPL) trace for line A. Red dotted line indicates a monoexponential fit to the experimental data.

Time-resolved microphotoluminescence (TRPL) measurements were carried out for the A line to characterize the emission kinetics affecting the properties of SPS, in particular, the fundamental limit for maximal photon generation rate. The TRPL trace is presented in Figure 3. 

The photoluminescence lifetime and its accuracy determined from the fit with a monoexponential decay (red dotted line in Figure 3) is (1.78 ± 0.02) ns, which is similar to the values reported for other InAs on InP QD-like structures [20,51,60,63,68,69,70]. The measurement was performed at low excitation power (0.1 µW) to minimize the probability of occupation of higher energy states, which would affect the PL lifetime. Therefore, the obtained PL decay time approximates well the radiative lifetime, which corresponds to the maximal single-photon emission rate of 0.5 GHz. However, this is just the upper limit, which is usually not achieved in the final SPS device due to the finite extraction efficiency of the emission, nonideal internal quantum efficiency of the emitters, as well as any other losses of carriers within the structure.

**Figure 4:**
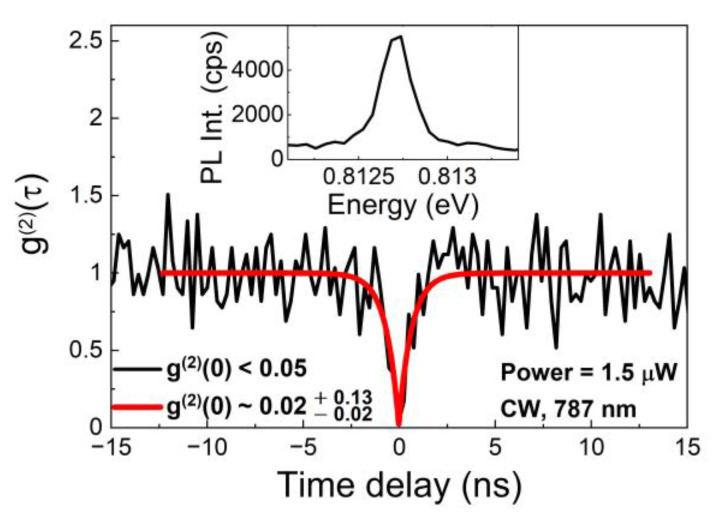
Second-order autocorrelation function *g*^(2)^(τ) for QDash emission line A under continuous-wave excitation (black line) and fit with Equation (1) (red line). Inset: corresponding microphotoluminescence spectrum.

To characterize single-photon emission properties of the investigated nanostructures, the second-order autocorrelation function *g*^(2)^(τ) was measured with CW excitation (787 nm) with the result presented in Figure 4.

The inset shows the corresponding microphotoluminescence spectrum recorded directly in the correlation setup. The autocorrelation function is obtained from the measurements of the coincidences’ histogram (time correlation) of the emission events on the two single-photon detectors in the Hanbury Brown and Twiss configuration. The measured curve is normalized by the mean number of coincidences for long time delays (outside the range close to the zero time delay between the emission events), where no time-correlation is expected between the photons and, therefore, Poissonian statistics of emission corresponding to *g*^(2)^(0) = 1 is expected. The obtained as-measured *g*^(2)^(0) value below 0.05 indicates a very low probability of multiphoton emission events without correction to compensate for the finite temporal resolution of the experimental setup, which would further lower the *g*^(2)^(0) value. The fitting was performed with the function, Equation (1):

(1) $$g^{\left(2\right)}\left(\tau \right)=1-\left[1-g^{\left(2\right)}\left(0\right)\right]e^{\frac{\left|\tau \right|}{t_{rise}}}$$

resulting in the final value of $g^{\left(2\right)}\left(0\right)\approx 0.02_{-0.02}^{+0.13}$. This is a very promising result and the best *g*^(2)^(0) value for QDashes emitting in the third telecommunication window obtained so far [54,55]. The demonstrated value of *g*^(2)^(0) is also comparable with the best results for symmetric quantum dots based on InP substrates emitting in this spectral range [51,59,61], however, it can still be improved to reach record values [12]. The presented results indicates that the investigated system with QDashes is fairly competitive for an efficient fiber network SPS.

**Figure 5:**
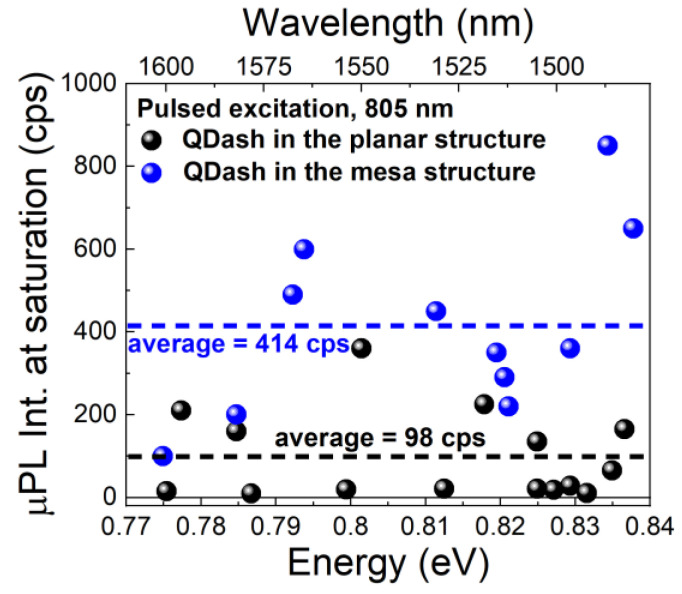
Comparison of µPL emission intensity at saturation from single QDashes in the planar structure (black) and in the mesa (blue).

Making mesas on a DBR structure provides emission directionality resulting from the photonic confinement. However, the formed 3D cavity is expected to have a very low finesse [33], so in the first approximation, the possible Purcell effect can be neglected, which is consistent with our photoluminescence lifetime results, i.e., the lifetimes measured for the planar structure (~1.9 ns) and for different types of photonic structures with QDashes (including mesas) are comparable. Figure 5 shows emission intensity values at saturation conditions (pulsed excitation) for a large number of QDashes in cylindrical mesas and for a set of QDashes in unpatterned area of the sample.

The average emission intensity from dashes in the planar structure is 98 and 414 cps for QDashes within the mesa structure. It shows that even the unoptimized mesas can already provide approximately fourfold increase in the emission intensity.

Although the photonic structures had not been optimized with respect to the extraction efficiency, its value was experimentally determined, in order to verify the current efficiency with the theoretical predictions for this kind of cylindrical photonic structures on a DBR in InP-based material system. The measurements were carried out with nonresonant (805 nm) pulsed excitation and with single-photon detectors. Based on that, µPL intensity was determined at saturation (excitation power providing the maximum probability of photon emission from QD after excitation) with ~80 MHz repetition pulsed excitation. As a result, emission extraction efficiency was estimated to be (1.0 ± 0.2)% for the best line from mesa structure of ~0.9 µm diameter and ~0.7 µm height. This value was obtained by considering the setup collection efficiency, including transmission of the microscope objective with NA = 0.4 (40%), transmission of the detection setup (5.5%), and the detector sensitivity (50%).

For such mesas, numerical simulations based on a finite element method were performed in JCMsuite solver for Maxwell equations [71,72] in order to estimate the extraction efficiency of emission from a dipole-like emitter imitating a quantum dot. Here, 100% of QDash internal quantum efficiency was assumed, namely, nonradiative decay rate is neglected. By considering the cylindrical mesa geometry of the exact dimensions as those investigated by µPL, and within a numerical aperture of 0.4 of the collected far-field emission as in the experiment, the extraction efficiency of 0.8% was obtained, which is consistent with the measured value.

**Figure 6:**
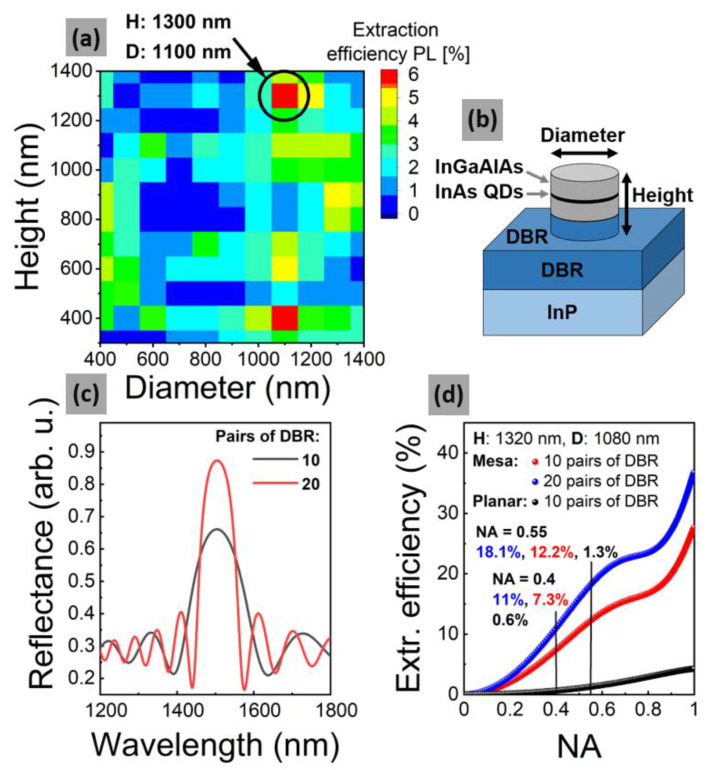
(**a**) Numerically determined emission extraction efficiency in the function of diameter and height of mesa structures at 1.5 µm. (**b**) Scheme of the mesa structure geometry. (**c**) Calculated reflection spectra for 10 and 20 pairs of distributed Bragg reflectors (DBR). (**d**) Numerically determined extraction efficiency in the function of numerical aperture of microscope objective for 10 and 20 pairs of DBR at 1.5 µm.

Moreover, in order to test the potential of this solution with respect to the extraction efficiency improvement, simulations for a wide range of mesa diameters (D) and mesa heights (H) were performed. Figure 6a shows that the design of the photonic structure can be further optimized only by modification of the mesa geometry—see Figure 6b.

In the case of two points on the map with the highest values (H_1_: 1300 nm, D_1_: 1100 nm and H_2_: 400 nm, D_2_: 1100 nm), additional calculations of the emission extraction efficiency as a function of the numerical aperture and the number of pairs of the DBR structure were performed. Based on simulations with a denser numerical grid around the selected geometry of the highest extraction efficiency (H_1_, D_1_), it is demonstrated that for a mesa of 1080 nm in diameter and 1320 nm in height, an extraction efficiency of approximately 7% for single-photon emission at 1.5 µm can be obtained. In addition, when more DBR mirror pairs are used to obtain higher reflectivity, it further improves the emission collection efficiency, as demonstrated in Figure 6c in accordance with calculated reflection spectra based on a transfer-matrix method [73]. Moreover, a numerical aperture higher than 0.4 can be used, e.g., to make a more direct comparison to the results presented in [63]. Finally, for a structure with 20 pairs of DBR and NA = 0.55, the resulting extraction efficiency is 18.1%, as shown in Figure 6d, what exhibits the real potential of the practical applicability of this type of SPS emitting in third telecommunication window. Besides, we observe a significant improvement in the extraction from 0.6% for a planar structure to 7.3% when taking into account the mesa structure (NA = 0.4), and it works similarly also for other numerical apertures, for instance, in the extreme case of NA = 1, the extraction efficiency of a planar structure is 4.3%, compared to 27.7% for a mesa, all proving that mesa structure significantly enhances the emission collection.

## 4. Conclusions

Optical properties of MBE-grown InAs/InGaAlAs/InP quantum dashes emitting in the telecommunication spectral range, placed in photonic confinement structures and on a distributed Bragg reflector, were investigated. The second-order autocorrelation function of single dash emission showed a clear antibunching dip with the as-measured value at the zero delay below 0.05. Emission intensity from dashes inside cylindrical mesas showed fourfold increase in intensity of emission, associated with the modification of the emission extraction efficiency, as compared to the planar sample. Based on pulsed-excitation measurements, the single-photon emission extraction efficiency to the first lens (numerical aperture of 0.4) of a free-space detection setup was determined in the range of (1.0 ± 0.2)%. The performed simulations indicated on the directions for further development and possible optimization steps of this kind of system to enhance the extraction efficiency well above 15% by just the mesa design and increasing the number of DBR pairs, increasing the application potential of this kind of structures as efficient and affordable single-photon sources.

## Data Availability

The data that support the findings of this research are available from the corresponding author upon reasonable request.
